# Coupled Stochastic Spatial and Non-Spatial Simulations of ErbB1 Signaling Pathways Demonstrate the Importance of Spatial Organization in Signal Transduction

**DOI:** 10.1371/journal.pone.0006316

**Published:** 2009-07-23

**Authors:** Michelle N. Costa, Krishnan Radhakrishnan, Bridget S. Wilson, Dionisios G. Vlachos, Jeremy S. Edwards

**Affiliations:** 1 Department of Chemical and Nuclear Engineering, University of New Mexico, Albuquerque, New Mexico, United States of America; 2 Department of Pathology, University of New Mexico Health Sciences Center, Albuquerque, New Mexico, United States of America; 3 Cancer Research and Treatment Center, University of New Mexico Health Sciences Center, Albuquerque, New Mexico, United States of America; 4 Department of Chemical Engineering, University of Delaware, Newark, Delaware, United States of America; 5 Molecular Genetics and Microbiology, University of New Mexico Health Sciences Center, Albuquerque, New Mexico, United States of America; Lund University, Sweden

## Abstract

**Background:**

The ErbB family of receptors activates intracellular signaling pathways that control cellular proliferation, growth, differentiation and apoptosis. Given these central roles, it is not surprising that overexpression of the ErbB receptors is often associated with carcinogenesis. Therefore, extensive laboratory studies have been devoted to understanding the signaling events associated with ErbB activation.

**Methodology/Principal Findings:**

Systems biology has contributed significantly to our current understanding of ErbB signaling networks. However, although computational models have grown in complexity over the years, little work has been done to consider the spatial-temporal dynamics of receptor interactions and to evaluate how spatial organization of membrane receptors influences signaling transduction. Herein, we explore the impact of spatial organization of the epidermal growth factor receptor (ErbB1/EGFR) on the initiation of downstream signaling. We describe the development of an algorithm that couples a spatial stochastic model of membrane receptors with a nonspatial stochastic model of the reactions and interactions in the cytosol. This novel algorithm provides a computationally efficient method to evaluate the effects of spatial heterogeneity on the coupling of receptors to cytosolic signaling partners.

**Conclusions/Significance:**

Mathematical models of signal transduction rarely consider the contributions of spatial organization due to high computational costs. A hybrid stochastic approach simplifies analyses of the spatio-temporal aspects of cell signaling and, as an example, demonstrates that receptor clustering contributes significantly to the efficiency of signal propagation from ligand-engaged growth factor receptors.

## Introduction

The ErbB family of receptors, under normal physiological conditions, regulate key cellular processes such as growth, proliferation and differentiation [1], [2], [3]. Overexpression of these receptors deregulates normal cellular function and is a contributing factor to tumorigenesis [4]. There are four members of the ErbB family (ErbB1, ErbB2, ErbB3 and ErbB4) and each family member has its own unique ligand specificity [5], kinase activity [2] and spatial organization on the membrane [1], [6]. In our current study, we have focused solely on the epidermal growth factor receptor (typically abbreviated ErbB1 or EGFR) and the ErbB1 activation of ERK, which is a mitogen activated protein kinase [7]. Ligand binding to ErbB1 stabilizes a conformation of the extracellular domain that allows receptor dimerization [8]. Dimerized receptors are active tyrosine kinases, capable of transautophosphorylation [8]. Phosphorylation of receptor cytoplasmic tails results in recruitment of SH2-containing adaptor and signaling proteins, such as Grb2, Sos, and Shc, that form a signaling scaffold to activate ERK [9].

Due to the importance of the ErbB1-activated ERK pathway, several ordinary differential equation (ODE) models have been developed to gain insight into this pathway [10], [11], [12], [13]. While ODE models have provided insight into the dynamics of this pathway, these models assume that the cell is a homogeneous well-mixed system. In other words, the ODE models neglect spatial localization and organization, such as membrane receptor clustering [3], [14]. Over the past decade, ODE models of the ErbB1-induced ERK pathway have evolved in complexity, becoming both larger and having more experimentally constrained parameters [15]. The first ErbB1/EGFR model was introduced in 1996 and had 35 reactions [16], whereas the most complete models available contain hundreds of reactions [10], [15].

The question remains whether these well-mixed deterministic models are capable of quantitatively describing the temporal dynamics of signaling, since there is significant evidence that cell membrane organization promotes the formation of localized “signaling platforms” [17], [18], [19], [20]. Major advances in our understanding of the membrane have led to a revision of the original Fluid Mosaic model (Singer and Nicholson, 1972), to a more ordered structure with distinct membrane microdomains of lipids and proteins [21], [22], [23] Advanced microscopy techniques have demonstrated that membrane properties, such as transient confinement zones and corrals, may restrict and govern the spatial-temporal dynamics of lipids and membrane proteins [24], [25], [26], [27], [28], [29]. The challenge is to develop computational approaches that can account for membrane spatial heterogeneity and evaluate the impact on signal propagation.

Spatial modeling has been implemented in many scientific disciplines, including physics, material sciences, chemistry, engineering and biological systems. However, the modeling methodologies used vary, with typical approaches including partial differential equations [30], agent-based modeling [31] and spatial Monte Carlo (MC) methods [32], [33], [34]. Spatial MC platforms are particularly powerful numerical simulation tools for studying the dynamics of membrane components [35], [36], [37], [38]. The application of spatial MC methods has been implemented by our group [36] to study ErbB reaction/diffusion and herein to study the effect of spatial heterogeneity on signal propagation. We report the development of a new computational framework that merges a spatial kinetic Monte Carlo (SKMC) algorithm for modeling reaction and diffusion events on the membrane with a stochastic simulator algorithm (SSA) [39] for modeling cytosolic reactions. This new algorithm, the Coupled Spatial and Non-spatial Simulation Algorithm (CSNSA), has enabled us to determine the effects that receptor clustering has on the initiation of signaling.

## Results

### Establishing Parameters for the Spatial Model

**Figure 1:**
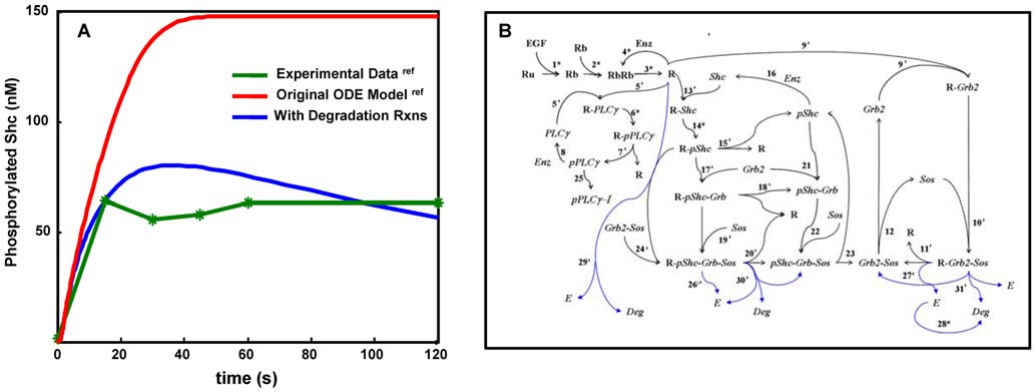
Parameter optimization and summary of reaction network. A) Optimization of modeling parameters based upon sensitivity analysis and ODE solution. Green line: Kinetics of Shc phosphorylation in EGF-stimulated hepatocytes (20 nM EGF) as determined by Kholodenko et al. [12]. Red line: results obtained using the ODE model of [12]. Blue line: improved fit of ODE solution to experimental data after incorporation of receptor degradation reactions. B) Summary of reaction network in the ODE and CSNSA models. Note that, in the spatial CSNSA model, stars mark membrane reactions handled by the spatial stochastic Monte Carlo algorithm. All remaining reactions are governed by the Gillespie algorithm. Additional reactions that were added to the original ODE model from Kholodenko et al. [12] are shown in blue. Numbering of reactions is arbitrary.

One goal of our study was to evaluate whether simulation results from a spatial stochastic model would differ significantly from a deterministic solution that assume all components are well-mixed. As a starting point, we began with the original ODE model developed by Kholodenko and colleagues [12]. We noted, however, that the ODE model produced results that deviated from the same group's experimental data [12]. We performed a sensitivity analysis to identify the most important enzymatic reaction parameters in the system. Based upon this analysis, we determined that incorporation of receptor degradation mechanisms results in a better fit to the experimental data (Figure 1A) and we fit the new parameters using the PottersWheel MatLab toolbox [40]. Additional reactions added during our model development are illustrated in blue within Figure 1B and the entire set of reaction parameters are summarized in Table 1. Our model modifications are consistent with other models that include negative feedback reactions [10], [11], [13]. In addition, it is noteworthy that the new parameters fit using the ODE model were not explicitly dependent on receptor diffusion. Appendix S1 describes our analytical approach to demonstrate the validity of this fit.

**Table 1:** Definition of the reactions in the spatial-temporal simulations.

Reactions	Rate Constants	
**Membrane Reactions**		
1. EGF+Ru ↔ Rb	Kf = 0.003	Kb = 0.06
2. Rb+Rb ↔ RbRb	Kf = 0.01	Kb = 0.1
3. RbRb ↔ R	Kf = 1	Kb = 0.01
4. R → RbRb	Vmax = 268	Km = 56.2
5. R-Sh ↔ R-pSh	Kf = 6	Kb = 0.06
6. R –PLCγ ↔ R –pPLCγ	Kf = 1	Kb = 0.05
**Interfacial Reactions**		
1. R+Shc ↔ R-Sh	Kf = 0.09	Kb = 0.6
2. R-pSh ↔ R+pShc	Kf = 0.3	Kb = 9×10^−4^
3. R-pSh+Grb2 ↔ R-pSh-G	Kf = 0.003	Kb = 0.1
4. R-pSh-G ↔ R+pSh-G	Kf = 0.3	Kb = 9×10^−4^
5. R-pSh-G+Sos ↔ R-pSh-G-	Kf = 0.01	Kb = 2.14×10^−2^
6. R-pSh-G-S ↔ R+pSh-G-S	Kf = 0.12	Kb = 2.4×10^−4^
7. R-pSh+G-S ↔ R-pSh-G-S	Kf = 0.009	Kb = 4.29×10^−2^
8. R+Grb ↔ R-G	Kf = 0.003	Kb = 0.05
9. R-G+Sos ↔ R-G-S	Kf = 0.01	Kb = 0.06
10. R-G-S ↔ R+G-S	Kf = 0.03	Kb = 4.5×10^−3^
11. R+PLCγ↔ R –PLCγ	Kf = 0.06	Kb = 0.2
12. R –pPLCγ ↔ R+pPLCγ	Kf = 0.3	Kb = 0.006
13. R-pShGS → R-pShGS+E	Kf = 8	
14. R-GS → R-GS+E	Kf = 48	
15. R+E → Deg+E	Vmax = 4.7	Km = 82
16. R-pShGS+E → Deg+E+pShGS	Vmax = 7560	Km = 78
17. R-GS+E → Deg+E+GS	Vmax = 5520	Km = 7560
**Cytosolic Reactions**		
1. G-S ↔ Grb2+Sos	Kf = 1.5×10^−3^	Kb = 10^−4^
2. pShc → Shc	Vmax = 2.4	Km = 14.2
3. pShc+Grb2 ↔ pSh-G	Kf = 0.003	Km = 0.1
4. pSh-G+Sos ↔ pSh-G-S	Kf = 0.03	Kb = 0.064
5. pSh-G-S ↔ pSh+G-S	Kf = 0.1	Kb = 0.021
6. pPLCγ → PLCγ	Vmax = 2	Km = 13
7. pPLCγ ↔ pPLCγ-I	Kf = 1	Kb = 0.003
8. E → Deg	Kf = 248	

Initial concentrations (nM) are Ru (varied), EGF = 20.42Vol_Extracellular_ (Vol_Extracellular_ is the volume of the cell (diameter of 20 µm) multiplied by the ratio of the volume of incubation medium per cell over the cytoplasmic water volume ∼33.3), PLCγ = 105, Grb2 = 85, and Sos = 34. First and second-order rate constants are in units of s^−1^ and nM^−1^ s^−1^ and the Michaelis-Menten constants Km and Vmax are in units of nM and nM s^−1^, respectively. Reactions are categorized as membrane reactions (handled by the SKMC), interfacial reactions (cytosolic species associating or dissociating with receptor) handled by the SKMC, and cytosolic reactions (handled by the SSA).

### Validating the CSNSA hybrid approach

**Figure 2:**
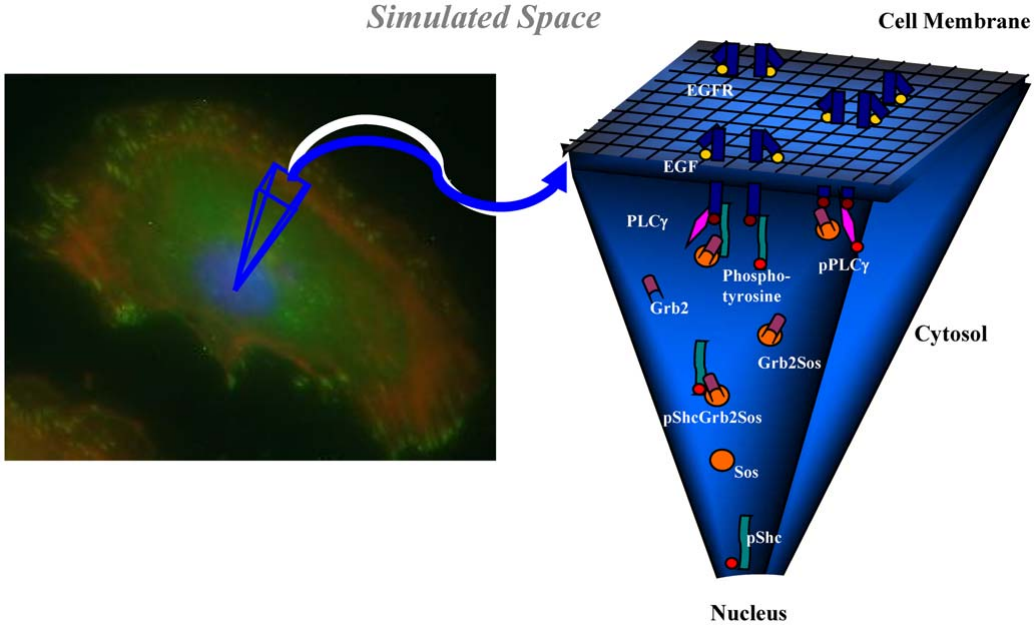
Illustration of the simulated space of the cell, consisting of two distinct domains: the cell membrane and the cytosol. The CSNSA model incorporates a Monte Carlo approach to simulate receptor diffusions and interactions on the cell membrane and couples to a spatial stochastic algorithm (Gillespie) for all cytosol interactions.

**Figure 3:**
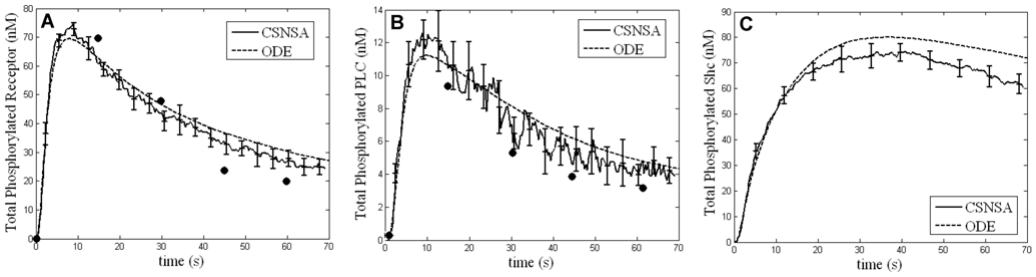
Comparison of the CSNSA and ODE solutions for receptor phosphorylation, PLCγ and SHC recruitment following EGF stimulation. Simulated kinetics of ErbB1 phosphorylation (A), PLCγ recruitment (B) and Shc phosphorylation after EGF (20 nM) using the ODE model (dashed lines) or the CSNSA model (solid black line). Results (A,B) from both simulation methods compare well with experimental data (solid circles) reported by Kholodenko et al. [12]

The novelty of the *CSNSA* approach lies in its computationally efficient framework that considers receptor diffusion and reaction in the 2-dimensional confines of the plasma membrane, while cytosolic reactions occur stochastically under well-mixed conditions. The simulated space is illustrated in Figure 2, with a full description of the CSNSA algorithm in the Methods section below. As an initial test, results were compared with the ODE solution (as described in Figure 1) and the experiment results in Kholodenko et al [12]. The simulation space was populated with an initial random distribution of receptor at a density of 141 receptors per µm^2^, each diffusing at 1×10^−14^ m^2^s^−1^ [41]. In both ODE and CSNSA models, reactions were initiated by addition of EGF ligand (20 nM). Results show that, when receptors are randomly distributed, the two approaches give similar results for the rate and extent of ErbB1 phosphorylation and for the recruitment of PLCγ (Figure 3). The CSNSA model predicts a slightly lower peak value and less sustained recruitment of Shc (Figure 3) when compared to the ODE solution. These results emphasize that the CSNSA hybrid stochastic model is comparable to deterministic solutions in the absence of local concentration gradients or membrane inhomogeneities.

### Predicting the Impact of Receptor Density vs. Clustering

**Figure 4:**
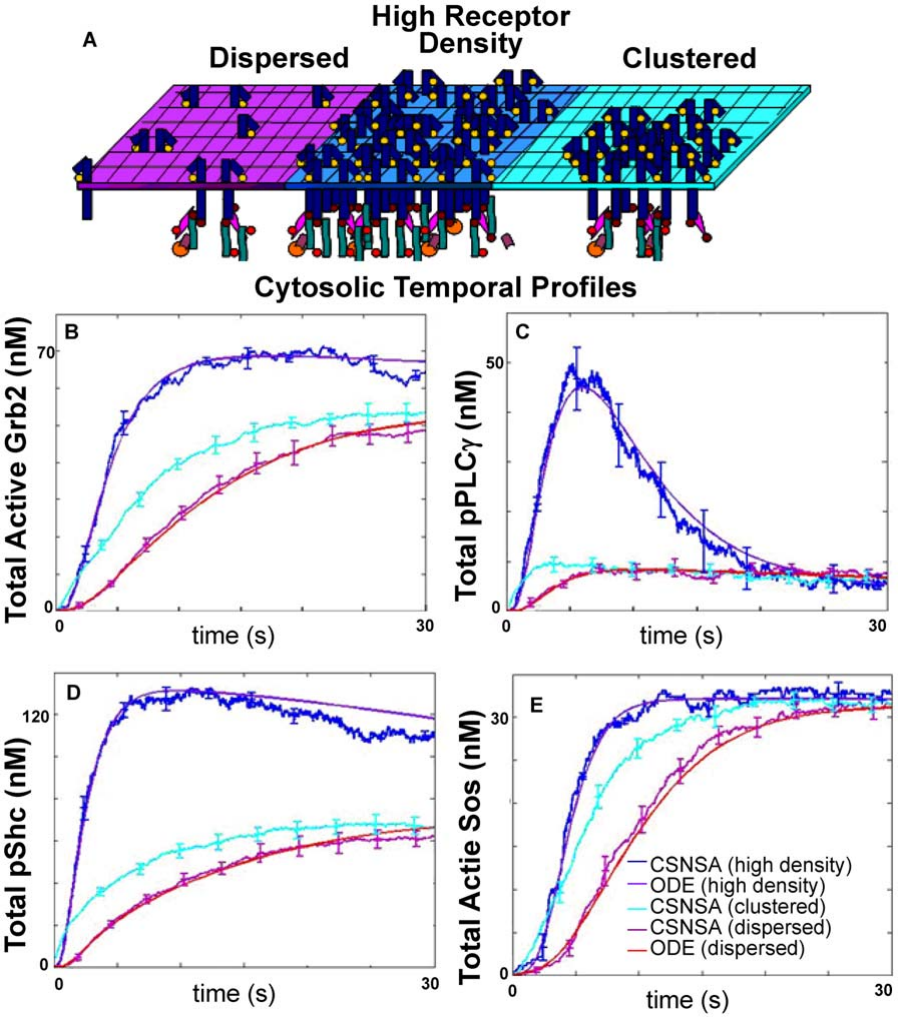
The spatial model predicts that receptor clustering enhances signaling efficiency by creating locally high receptor densities. A) Schematic illustration of three simulation cases: dispersed (left), high-receptor density (middle), and highly clustered (right). See legend for key to colored lines in each plot. Results predict the kinetics of Grb2 activation (B), PLCγ phosphorylation (C), Shc phosphorylation (D) and Sos activation (E). Active Grb2 is equivalent to: RGrb2+RGrb2Sos+RpShcGrb2+RpShcGrb2Sos+Grb2Sos+pShcGrb2+pShcGrb2Sos; Total phosphorylated PLCγ = RpPLCγ+pPLCγ+pPLCγI; total phosphorylated Shc = RpShc+RpShcGrb2+RpShcGrb2Sos+pShc+pShcGrb2+pShcGrb2Sos; total Sos RGrb2Sos+RpShcGrb2Sos+Grb2Sos+pShcGrb2Sos.

We next used the CSNSA to determine the effects of receptor spatial distribution and density on downstream signaling. We defined three different conditions, as shown in the schematic of Figure 4. In the first condition (magenta), the simulation space contained a modest density of dispersed receptors (106 receptors per µm^2^). In the second condition (dark blue), the simulation space contained a high density of well dispersed receptors (705 receptors per µm^2^). The final simulation condition (cyan) began with a dense cluster of receptors, which was initially confined to a central region of 705 receptors per µm^2^ and then permitted to diffuse over time to encompass the entire simulation space for a final density of 106 receptors per µm^2^. For each regime we examined how initial receptor density and clustering conditions influenced coupling to four of ErbB1's adaptor proteins. The temporal profiles of the cytosolic species Grb2, Sos, and pShc and membrane-bound PLCγ are shown in Figure 4B–E.

All temporal profiles of the CSNSA were compared with their ODE solutions (shown in purple and red). The most notable differences came from the clustered regime (cyan), which had the same receptor concentration of 106 receptors per µm^2^ as the non-clustered regime (magenta) but was initially confined to a smaller region. The clustered regime showed a marked increase in the amplitude of signal propagation in comparison to the ODE solution. These data demonstrate that spatial models are needed to accurately predict the consequence of membrane heterogeneity on signal propagation and set the stage for more refined considerations of signaling platforms.

## Discussion

In this work, we describe a new, efficient computation framework for evaluating the contributions of spatial organization to important cellular processes. Although applied here to study ErbB1 signal initiation at the plasma membrane, the algorithm should be readily adaptable to other receptor systems, organelle sites and biochemical cascades. We show that, when considering well-mixed systems, solutions obtained using the CSNSA hybrid model and the more traditional ODE solutions are comparable. However, given the growing evidence for membrane compartmentalization at both the plasma membrane and internal organelles [6], [42], [43], we propose that the spatial stochastic model will more accurately predict the outcomes of events that take place between membrane proteins and lipids and their cytosolic binding partners.

As an example, we used CSNSA to demonstrate that receptor clustering creates a more efficient signaling environment. The existence of receptor clusters is well established [23], [44], [45], but the significance of this membrane organization has been approached in only a few recent publications [31], [46]. Our previous work concluded that ligand-independent ErbB1 dimerization is likely to be dependent on two factors: density and the probability of receptor “fluxing” from a closed (dimerization-incompetent) to an open (dimerization-competent) conformation [31], [47]. Because clustering creates locally high receptor concentrations, it enhances the probability for collision between receptors that are transiently in the conformationally “open” state [31]. Here, we show that ErbB1 clustering also enhances the signaling output of receptors, based upon the more efficient recruitment of PLCγ1, Grb2, Sos and Shc.

The importance of spatial effects is emerging as an important topic in systems biology, with technologies such as single particle tracking and electron microscopy demonstrating unique spatial domains [25], [26], [48], [49], [50], [51], [52]. In this work, we applied a novel algorithm to show a direct link between spatial heterogeneity and downstream signaling. We propose that future studies of receptor signaling should seek to gain a fundamental understanding of the spatial interactions and spatial organization of the receptors and to apply these concepts to predictions of signaling output. ErbB receptor clustered domains have been observed in many cancers using different microscopy techniques [6], [44]. Understanding this bigger picture of spatial-temporal protein interactions will drive forth knowledge of cell signaling events and offer the potential to lead towards better drug treatment options.

## Methods

### Coupled Spatial, Non-spatial Simulation Algorithm (CSNSA)

**Figure 5:**
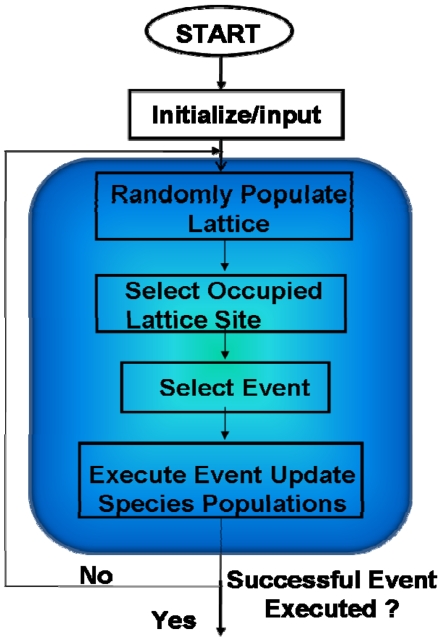
The spatial kinetic Monte Carlo algorithm, as implemented in the *CSNSA*. This algorithm differs from the original algorithm of Mayawala et al [46] in the time update, which occurs recursively until a successful event is selected. Time is not updated when a null event occurs. A detailed description is provided in the text.

The Coupled Spatial Non-spatial Simulation Algorithm, CSNSA, is a hybrid model that considers the diffusive behavior and organization of receptors and other membrane components within a 2-D framework, bordered by a well-mixed cytosol. A spatial kinetic Monte Carlo algorithm was employed to capture the spatial-temporal dynamics of receptors on the cell membrane [36] (Figure 5); we used a null-event algorithm that allows ease of implementation and variation of the underlying model. For computational simplicity, the cytosol is treated as a well-mixed solution and modeled with the stochastic simulation algorithm of Gillespie [39]. This assumption is reasonable in the cytosol, given that the diffusivity of proteins in the cytosol (1×10^−10^ m^2^s^−1^) [53] is four orders of magnitude larger than that in the plasma membrane (1×10^−14^ m^2^s^−1^) [41].

The two algorithms are coupled using the CSNSA, which employs a novel algorithm that selects and executes reactions that allow the molecular species to evolve in space and time. The coupling method takes into account the stochastic nature of biological systems. The first step of the CSNSA is to select a spatial domain (cell membrane or cytosol) and thus the corresponding algorithm for the next event. The selection is made by computing the probabilities of a membrane (SKMC) event or a cytosolic (SSA) event, which are calculated as:
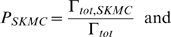


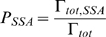
where 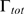 is defined as,



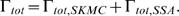



The total transition rate for the SKMC, 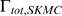, is the sum of all transition rates for all SKMC events, or more specifically the transition rate for diffusion (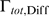) and the sum of the reaction events (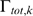) for all 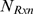 reaction types, 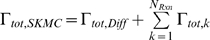, where 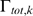
is the total transition rate for each reaction type defined over all lattice sites 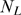, 
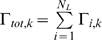
. 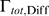 is defined as the sum of the transition diffusion rate 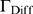 over all lattice sites 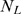, 
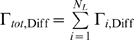
. Thus, 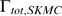 is defined as:
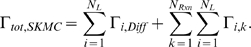



The SSA only accounts for stochastic variations in species populations and does not consider the spatial organization in the cytosol, and therefore does not contain a diffusion term. The 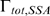 is defined as the sum of 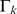 over all reaction types, 
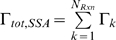
.

**Figure 6:**
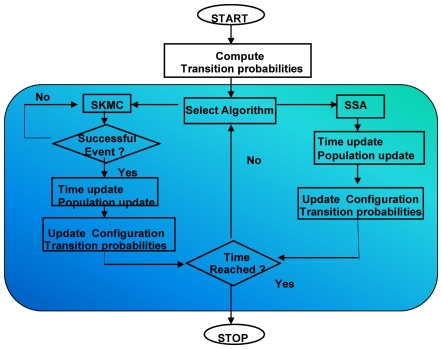
Schematic of *CSNSA*. Coupled Spatial Nonspatial Simulation Algorithm, *CSNSA*, combines the spatial stochastic algorithm [39] depicted in the right branch, with the spatial kinetic Monte Carlo algorithm [56] in the left branch. Upon selection of a branch, a successful event has been executed, species populations are updated, transition rates and probabilities are recomputed, and time advances. The *CSNSA* is described in greater detail within the text.

**Figure 7:**
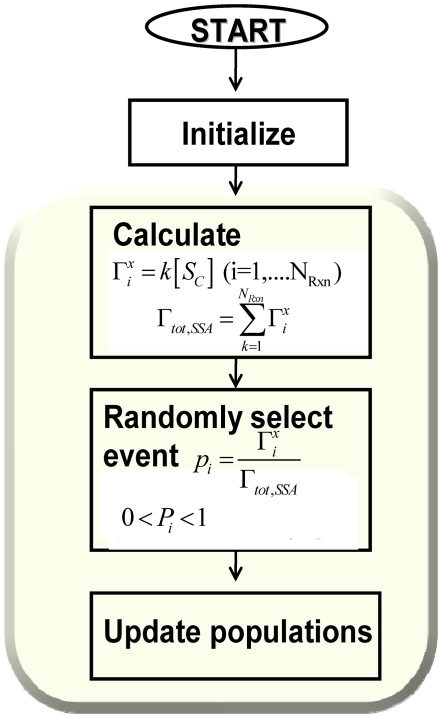
Schematic of the SSA algorithm, as coupled to the hybrid algorithm. This algorithm is used for all cytosolic interactions. Being a rejection free algorithm, a successful event (reaction) is chosen and executed in each iteration.

The combined MC method operates like a single MC method by considering the superposition of all processes. Time is updated in a “combined” manner from 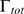 with an average time step as, 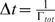. Given that the two algorithms are different (null-event vs. rejection free), the CSNSA is a hybrid method. In order to properly match time scales, upon selection of a spatial event, the SKMC model is continuously executed until a successful event is selected, as shown in Figure 6, based on probability theory described in [33]. The complete algorithm, which is shown in Figure 7, was implemented in Fortran 90. Since the algorithm is stochastic, 10 simulations with different seeds for the random number generator were used. The *CSNSA* was benchmarked by comparison to an ODE model in a reaction-limited system, where the diffusion coefficient in the CSNSA was made fast compared to the reaction rates (Figure 4). The typical CPU time for 50 receptors/lattice is ∼15 min, for 125 receptors/lattice is ∼2880 min, and for 500 receptors/lattice is ∼14400 min on an Intel® Xeon™ CPU 3.2 GHz processor with 8.00 GB of Ram.

### Spatial Kinetic Monte Carlo (SKMC)

Once an algorithm is selected and executed, transition probabilities are computed again at each time step. Computing 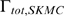 involves computing the 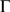 values for the SKMC over the entire lattice. This computation is the most CPU intensive step in the simulation algorithm. We, therefore, used an optimized computation method. In order to maximize efficiency, a local region that is affected by the previous reaction event is defined [36], and the 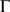 for each lattice site is computed for this region both before and after the event has been executed. This eliminates scanning the entire lattice before and after an event is implemented, and the new 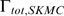 is calculated by:
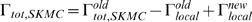
where, 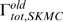 is the total transition probability computed initially or at a previous successful MC event, 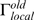 is the sum of transition probabilities of all sites affected by an executed event based on the old configuration, and 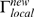 is the sum of transition probabilities of all sites affected by an executed event based on the new configuration.

The SKMC algorithm is a modified null-event lattice MC method; for further details see Mayawala et al. [36]. All reactions that are on the lattice or reacting with a species on the lattice are handled by the SKMC (see Figure 2, * denotes membrane reactions and ` denotes interfacial reactions). These reactions include ligand association and dissociation, receptor dimerization and decomposition, receptor phosphorylation and dephosphorylation, and phosphorylated receptor associating with and disassociating from cytosolic species. When an interfacial reaction occurs, a molecule of the cytosolic species is subtracted from the cytosolic population and the membrane species is converted to a new species at the same location on the lattice.

The spatial domain is a two-dimensional lattice with periodic boundary conditions. The initial condition of the lattice is dependent on user specifications and can either be randomly populated or clustered in pre-defined domains. The algorithm is implemented by selecting an occupied lattice site, choosing a successful (reaction or diffusion) or unsuccessful (null) event based on the probabilities, and if a successful event was chosen, executing the event.

An event is selected by computing the probability distribution for all events, defined as: 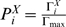, for lattice site *i* and event *x*. Table 2 shows the events executed by this algorithm and the equations for computing Γ^X^ for each event. Γ_max_ is defined as 
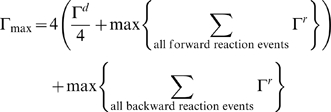
where the multiple of four accounts for events occurring in each of the four directions on the square lattice.

**Table 2:** Membrane Microscopic Events and Transition rates.

Microscopic Event	Transition Rate
**Diffusion**	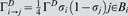 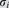 is the occupancy(discrete) that is 1, if site 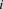 is filled, and 0, if site 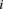 is empty (a single index indicating the site is used to simplify notation). 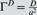 , where 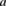 is the microscopic lattice pixel dimension taken equal to the encounter radius, and *D* is the diffusivity of a receptor *B_i_* denotes the set of sites to which diffusion from site *i* can occur which includes all 4 first-nearest neighboring sites
**Reactions**	
Ligand Association Reaction (S_L_+M → M*)	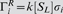 *k* is the macroscopic reaction rate constant with units as [s^−1^]
Ligand Disassociation Reaction (M* → S_L_+M)	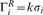 *k* is the macroscopic reaction rate constant with units as [s^−1^]
Dimerization Reaction (M*+M* → D)	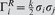 *k* is the macroscopic reaction rate constant with units as [(receptors/sites)^−1^ s^−1^]
Decomposition Reaction (D → M*+M* )	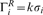 *k* is the macroscopic reaction rate constant with units as [s^−1^]
Phosphorylation/Dephosphorylation Reaction (D ↔ pD)	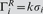 *k* is the macroscopic reaction rate constant with units as [s^−1^]
Cytosolic Association Reaction	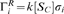 *k* is the macroscopic reaction rate constant with units as [s^−1^]
Cytosolic Disassociation Reaction	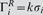 *k* is the macroscopic reaction rate constant with units as [s^−1^]

Γ is defined on a square lattice with lattice species M, monomers, D, dimers, and pD, phosphorylated dimers. Sx are species either within the cytosol SC or in the extracellular space SL. Details are provided in the text.

The spatial algorithm is coupled with the Stochastic Simulation Algorithm (SSA); therefore, unlike the original SKMC algorithm [36], the *CSNSA* is recursive in that it continuously selects an event until a successful event is chosen and executed as shown in Figure 6; therefore time is not updated if an unsuccessful event is selected.

### Stochastic Simulation Algorithm (SSA)

The non-spatial SSA developed by Gillespie [39] was used to model protein association reactions in the cytosol. The algorithm begins with initializing species populations and time; then propensities for all reactions are computed, and an event is randomly selected and the time is updated. This is a rejection free method; therefore, a reaction event is chosen and time is updated by an increment whose average is 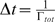.

### Interfacial Reactions

Interfacial reactions occur when a cytosolic species binds to or detaches from a receptor on the square lattice. In the former case, a molecule from the cytosolic species is subtracted from the cytosol population and a new product is produced at the site that was previously occupied by the reacting receptor. In the latter case, the converse procedure occurs. An example is shown in Table 1 (Interfacial Reaction #1), in which a cytosolic species, Shc, binds to a receptor, R, occupying site k producing product R-Shc at site k.

The rate constants for cytosolic reactions are calculated by first computing the cytosolic volume (V_cyt_ = 1/3 rL^2^ µm^3^), where r is the radius of the cell, and L is the lattice dimension. Next we compute the number of molecules per µm^3^, N_sp_. By multiplying the product of V_cyt_ and N_sp_ with the rate constant (given in terms of molecules^−1^ s^−1^for bimolecular reactions or s^−1^ for unimolecular reactions), we obtain a transition rate with units of molecules s^−1^.

### Sensitivity Analysis

To elucidate a mechanism that agrees with the experimental results [12] and explains the biological nature of our system, we modified the reaction scheme developed by Kholodenko et al. [12]. A sensitivity analysis was performed on the reaction mechanism, using the decoupled direct method and the backward differentiation formula method, as implemented in the NASA Glenn chemical kinetics and sensitivity analysis code LSENS [54], [55]. In addition to the species concentrations, these methods automatically follow the temporal evolution of the first-order sensitivity coefficients d*C*/dη*_j_*. The vector *C* contains the concentrations of all biochemical species and η *_j_* is a parameter of interest, such as an initial concentration or a rate constant. The parameters of the new system were refined, and fits were performed for the new reactions shown in blue in Figure 1 and for the Michaelis-Menten reactions using PottersWheel. The parameters to refine were determined to be sensitive using the LSENS package.

## Supporting Information

Appendix S1 (0.05 MB DOC)
