# Supplementary material for: Coupled Stochastic Spatial and Non-Spatial Simulations of ErbB1 Signaling Pathways Demonstrate the Importance of Spatial Organization in Signal Transduction
Source: PLoS One. 2009 Jul 23;4(7):e6316. doi: 10.1371/journal.pone.0006316 (PMC2710010; doi:10.1371/journal.pone.0006316)
Supplement: Appendix S1 — (0.05 MB DOC) [file pone.0006316.s001.doc]

**Appendix A**

The effective kinetic rate constant for diffusion-reaction problems derived by Lauffenburger and Linderman is

(1)

where $D_{AB}=D_{A}+D_{B}$, $D_{A}$and $D_{B}$are the diffusivities of A and B, $\rho_{A}$is the density of molecules of A (number of molecules per unit area), is one-half of the mean displacement between molecules of A distributed in a certain area, $k_{Areal}$is the intrinsic reaction rate constant in units of (receptors /area)^-1^ s^-1^, and s is the encounter radius. The relationship between $k_{Areal}$ and $k$ is given as, $k_{Areal}=ks^{2}$.

Using Eq 1. and combining it with the expression for resistance in a series model

$k_{PDE}=\left(\frac{1}{k_{Areal}}+\frac{1}{k_{Diffusion}}\right)^{-1}$ (2),

the diffusion-limited reaction rate constant, $k_{Diffusion}$is determined to be

$k_{Diffusion}=\frac{4\pi D_{AB}}{\ln\left(\frac{1}{\pi\rho_{A}s^{2}}\right)}$ (3).

for diffusion limited cases, $k_{Diffusion}<<k_{Areal}$, $k_{PDE}\approx k_{Areal}$as was the case for the fitted parameters in this work. In diffusion limited cases, $k_{PDE}$is the fitted parameter and can be related back to the ODE parameter using Eq. 1. Using this approach, small differences were found in enzymatic reactions leading to the conclusion that omission of diffusion-limited contributions contributed negligible effects on the model.

.
